# Reversible Self-Healing Carbon-Based Nanocomposites for Structural Applications

**DOI:** 10.3390/polym11050903

**Published:** 2019-05-17

**Authors:** Liberata Guadagno, Luigi Vertuccio, Carlo Naddeo, Elisa Calabrese, Giuseppina Barra, Marialuigia Raimondo, Andrea Sorrentino, Wolfgang H. Binder, Philipp Michael, Sravendra Rana

**Affiliations:** 1 Department of Industrial Engineering, University of Salerno, Via Giovanni Paolo II 132, 84084 Fisciano, Italy; lvertuccio@unisa.it (L.V.); cnaddeo@unisa.it (C.N.); elicalabrese@unisa.it (E.C.); gbarra@unisa.it (G.B.); mraimondo@unisa.it (M.R.); 2 Institute of Polymers, Composites and Biomaterials (IPCB-CNR), via Previati n. 1/E, 23900 Lecco, Italy; andrea.sorrentino@cnr.it; 3 Macromolecular Chemistry, Institute of Chemistry, Faculty of Natural Science II, Martin Luther University Halle-Wittenberg, Von-Danckelmann-Platz 4, 06120 Halle, Germany; wolfgang.binder@chemie.uni-halle.de (W.H.B.); philipp.michael@chemie.uni-halle.de (P.M.); srana@ddn.upes.ac.in (S.R.); 4 Department of Chemistry, University of Petroleum and Energy Studies (UPES), Bidholi Dehradun 248007, India

**Keywords:** smart materials, carbon–carbon composites (CCCs), thermosetting resins, mechanical properties, supramolecular interactions

## Abstract

Reversible Hydrogen Bonds (RHB) have been explored to confer self-healing function to multifunctional nanocomposites. This study has been carried out through a sequence of different steps. Hydrogen bonding moieties, with the intrinsic ability to simultaneously perform the functions of both hydrogen donors and acceptors, have been covalently attached to the walls of carbon nanotubes. The epoxy matrix has been modified to adapt the formulation for hosting self-healing mechanisms. It has been toughened with different percentages of rubber phase covalently linked to the epoxy precursor. The most performant matrix, from the mechanical point of view, has been chosen for the incorporation of MWCNTs. Self-healing performance and electrical conductivities have been studied. The comparison of data related to the properties of nanocomposites containing incorporated functionalized and nonfunctionalized MWCNTs has been performed. The values of the electrical conductivity of the self-healing nanocomposites, containing 2.0% by weight of functionalized multiwalled carbon nanotubes (MWCNTs), range between 6.76 × 10^−3^ S/m and 3.77 × 10^−2^ S/m, depending on the nature of the functional group. Curing degrees, glass transition temperatures, and storage moduli of the formulated multifunctional nanocomposites prove their potential for application as functional structural materials.

## 1. Introduction

The concept of materials having the ability to repair themselves is mainly inspired by nature. In living systems, damages which do not completely compromise the structural entity of the system or part of it are able to activate spontaneous healing mechanisms. The big challenge to transfer this ability to structural synthetic materials lies in the fact that these materials, unlike living systems, have no metabolic activity. However, even in inanimate matter, nature provides effective insights to achieve this goal. The imitation of natural mechanisms opens emerging and fascinating perspectives. It could have a significant impact on the extent of the working life and safety of synthetic materials for several applications. Among synthetic materials, thermoplastic and thermosetting polymers are widely produced for their employment in many technological and industrial sectors; hence, the possibility to add self-repair functions to these materials is under investigation by researchers around the globe [1,2,3,4,5,6,7,8,9,10,11,12,13,14,15,16,17,18,19,20,21,22,23].

An efficient self-healing material must be able to regenerate its structural integrity without any external stimulus at the moment in which the damage happens [24,25], thus imitating the healing mechanisms of animated bodies. In soft materials with low values of glass transition temperature (lower than the service temperature of the material), high chain mobility allows different possible self-healing mechanisms. For load-bearing materials characterized by high stiffness and poor mobility of the chains, there are severe limits in the choice of the self-healing mechanisms and therefore limited chances of successful results. Recently, many alternative strategies to microencapsulated systems have been proposed, such as hollow fibers, microvascular networks [26,27], or mechanochemical approaches [28,29,30]. Among these new approaches, chain dynamics and aspects including supramolecular polymer chemistry have been investigated [31,32,33,34,35,36,37,38,39,40,41,42,43,44,45,46,47,48,49,50,51,52,53,54,55,56,57,58]. Thus, attractive interactions based on the cumulative effects of week interactions such as hydrogen bonds and π–π stacking interactions can play a crucial role in repetitively activating healing cycles [40,59,60]. The effects of weak noncovalent interactions on the material properties are often underestimated, especially when dealing with amorphous or crystalline solid materials. Weak interactions, even weaker than that of the hydrogen bond, can strongly influence the material properties and impart or reinforce functions of relevant technological interest.

It has been recently highlighted that hydrogen bonding combined with other weaker noncovalent interactions can strongly affect the geometry and, consequently, the properties of the perovskite crystals. For instance, Varadwaj et. al. evidenced the relevant role of hydrogen bonding and other noncovalent interactions in determining the octahedral tilting in a CH_3_NH_3_PbI_3_ perovskite semiconductor system [61]. Form this study, the authors concluded that it is incorrect to attribute an important role only to the strong noncovalent interactions because other noncovalent weak interactions also affect the geometrical parameters and hence the physical performance of the material.

In this paper, MWCNTs have been functionalized with the aim to develop multifunctional self-healing epoxy nanocomposites, considering the idea to design materials and structures with different integrated functionalities. In particular, electrically conductive nanoparticles (carbon nanotubes or graphene-based nanoparticles) currently embedded in resins to enhance the electrical conductivity of the resulting nanocomposites act as a support for functional groups able to simultaneously impart self-healing ability to polymeric matrices. Furthermore, the possibility to develop electrically conductive self-healing composites opens new interesting perspectives in the sector of the self-responsive materials. Indeed, smart functions such as self-sensing, anti/de-icing, self-curing, etc. can be integrated into the materials/structures by exploiting the intrinsic electrical characteristics of the materials. Furthermore, the incorporation of nanostructured forms of carbon in polymeric matrices can simultaneously enhance not only the electrical conductivity and self-responsive smart functions, but also other desirable properties, such as thermal resistance, flame resistance, and durability [62,63,64,65,66,67,68,69].

Particular attention has been paid to the modification of the thermosetting matrix to make it able to host transient bonds on which the healing function is based.

Moieties with the intrinsic ability to simultaneously behave as hydrogen donors and acceptors have been covalently attached to the walls of carbon nanotubes to allow the formation of RHB interactions. Hydrogen bonds, in fact, can act as reversible hooks, effectively enabling the opening and the closing of the hooks, thus allowing a dynamic reiteration of the self-repair events. An interesting feature for real industrial application is the possibility to activate repeated healing events even in the same zone. In fact, it is possible with this strategy to enable connections and reconnections and thus the formation of supramolecular networks with the only condition that the moieties able to establish hydrogen bonds must be at a suitable distance to sense the attractive interactions.

**Figure 1:**
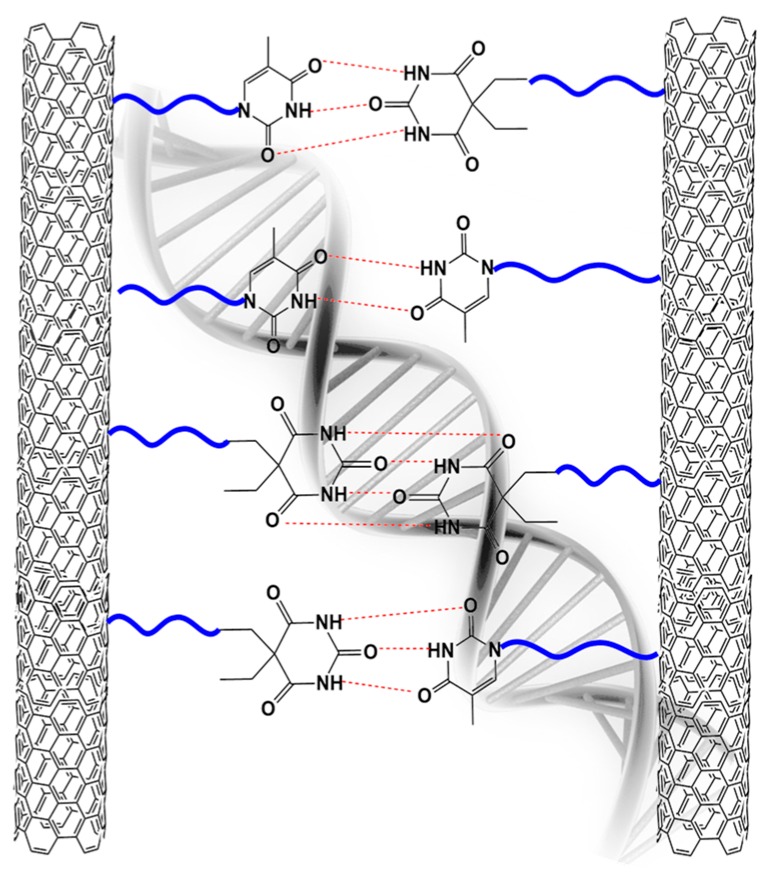
Example of hydrogen bonds in the inanimate matter between functional groups covalently bonded to the walls of multiwalled carbon nanotubes (MWCNTs).

The design of these reversible interactions is inspired by living systems, such as the remarkable capability of the DNA double helix to form, break (during the strand separation), and reform hydrogen bonds (see the schematic illustration of Figure 1). We have tried to imitate this great ability to form, break, and reform hydrogen bonds in inanimate matter between functionalized MWCNTs (as in Figure 1).

**Figure 2:**
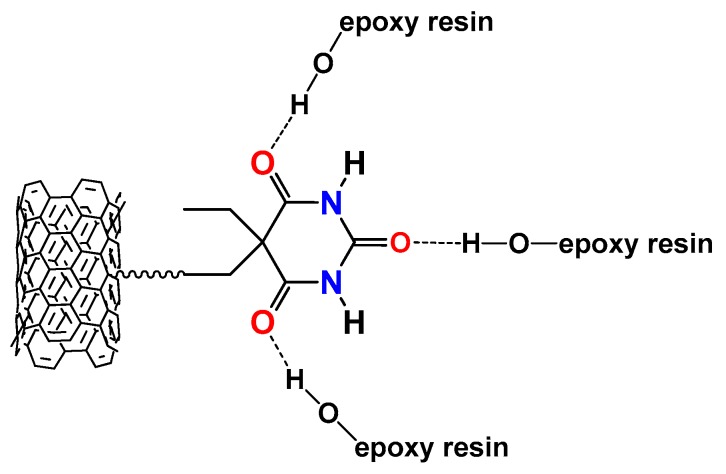
Example of hydrogen bonds between functional groups covalently bonded to the walls of MWCNTs and the –OH of the resin network.

Furthermore, it is expected that the presence of hydrogen bonding moieties on MWCNT walls is also able to establish RHB interactions with the –OH of the epoxy resins, as shown in the scheme of Figure 2.

The interactions shown in the scheme of Figure 2 are very likely, due to the relevant presence of –OH groups determined by the curing process.

## 2. Material and Methods

### 2.1. Materials

#### 2.1.1. Preparation of Functionalized MWCNTs

**Figure 3:**
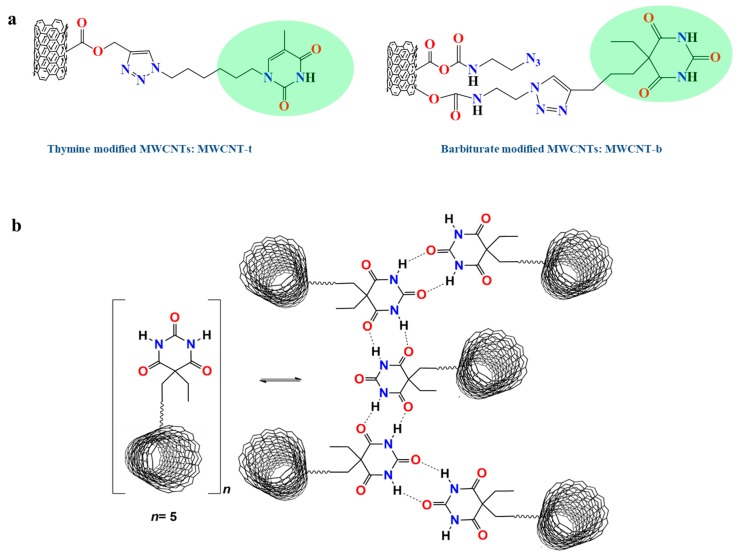
(**a**) Scheme representative of MWCNTs modified with thymine and barbituric acid-based functional groups; (**b**) Example of hydrogen bonds, which can be active during damage and healing events for barbiturate-modified MWCNTs.

Carbon nanotubes have been modified with thymine (MWCNT-t) and barbituric (MWCNT-b) acid-based moieties (see the scheme of Figure 3a) via copper(I)-catalyzed alkyne/azide cycloaddition (CuAAC) “click” reaction following a procedure already described in the literature [60]. An example of hydrogen bonds which can be active during damage and healing events for barbiturate modified MWCNTs.

#### 2.1.2. Modification of the Hosting Toughened Matrix

An aeronautical epoxy resin has been toughened using a fluid rubber carboxyl-terminated butadiene acrylonitrile (CTNB). The COOH groups of the liquid rubber react with the oxirane rings of the resin, tetraglycidyl methylene dianiline (TGMDA), to form covalent bonds. This reaction allows the dispersion of the rubber phase in the form of micrometric domains in the mixture of the initial epoxy precursor [60].

**Figure 4:**
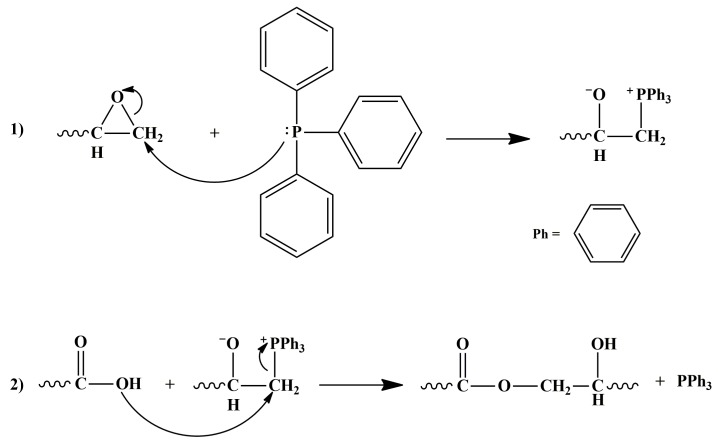
Scheme of the two steps reaction between the epoxy precursor TGMDA and the rubber phase CTNB: (**1**) nucleophilic attack of PPh_3_ catalyst on the carbon atom of the oxirane ring generating the phosphonium salt; (**2**) reaction between the carboxylic group of the CTNB copolymer and the phosphonium salt generating the TCT modified precursor.

Two epoxy mixtures were prepared by mixing the precursor TGMDA with 10 phr of triphenylphosphine (PPh_3_) and two different percentages 12.5 phr and 25.0 phr of CTNB, respectively. The triphenylphosphine promotes the reaction between the oxirane rings of the epoxy precursor and the carboxylic groups of the CTNB copolymer. The reaction mixtures were kept at 170 °C in an oil bath under mechanical agitation for 24 h. The product is indicated here as TCT and the reaction mechanism is shown in Figure 4 [60].

The mixture TCT was cooled at 120 °C. Afterward, a mixture here named TCTB was obtained by adding to the TCT mixture the diluent 1,4-butanedioldiglycidylether (BDE) at the ratio TGMDA/BDE of 80/20 by weight. Finally, 4,4′-diaminodiphenyl sulfone (DDS), was added to hardener the mixture TCTB, obtaining the epoxy mixtures TC12.5 TBD (for the formulation with 12.5 phr of CTNB) and TC25.0TBD (for the formulation with 25.0 phr of CTNB). A formulation without rubber phase, named TBD, was also prepared for comparison. All epoxy mixtures were kept under mechanical stirring at 120 °C for 1 h and then degassed in vacuum at 100 °C for 1 h. They were poured in the molds and solidified in an oven with a curing cycle composed of two isotherms: the first at 125 °C for 1 h followed by a second one at 200 °C for 3 h.

#### 2.1.3. Manufacturing of Multifunctional Self-Healing Nanocomposites

Functionalized carbon nanotubes (MWCNT-t and MWCNT-b) and nonfunctionalized MWCNTs have been embedded in different percentages (0.5 and 2.0% by weight) in the hosting toughened matrix with 12.5 phr of CTNB. Carbon nanotubes have been dispersed at 120 °C in the TCTB mixture, before the addition of the curing agent using an ultra-sonication process for about 20 min. Hielscher model UP200S (200 W, 24 kHz) (Hielscher Ultrasonics GmbH, Teltow, Germany) ultrasound was employed for this step. The developed self-healing nanocomposites are listed in Table 1.

### 2.2. Methods

#### 2.2.1. Thermo-Mechanical Characterization

DMA 2980 (TA instrument, Corp. 159 Lukens Drive New Castle, DE, USA) was used to perform the dynamic mechanical characterization. Temperature sweep test with the three points bending geometry was performed on specimens with dimensions 3 × 4 × 35 mm^3^. Samples have been tested in the viscoelastic region by setting the displacement amplitude at 0.1% and the frequency at 1 Hz. 

The measurements were performed with a heating rate of 3 °C/min over a temperature range of −50–300 °C after having carried out the calibration of the clamp compliance, force, and furnace temperature. Tan δ peak was taken as a measurement of the glass transition temperatures.

#### 2.2.2. Calorimetric Analysis

Differential Scanning Calorimetry analysis has been carried out to estimate the curing degree (DC). A Mettler DSC 822/400 (Mettler-Toledo Columbus, OH, USA) thermal analyzer was used.

The curing degree (DC) was calculated, considering that the exothermic heat (Δ*H*) during the dynamic heating is proportional to the extent of the curing reaction, from the total heat of reaction (Δ*H_T_*) of the uncured material and the residual heat of reaction (Δ*H*_Res_) of the partially cured epoxy resin according to Equation (1).

(1) $$\mathrm{DC}=\frac{\Delta H_{T}-\Delta H_{\mathrm{Res}}}{\Delta H_{T}}\times 100$$

In order to determine the total heat of reaction (Δ*H_T_*), the thermal analysis was performed on uncured formulations. The samples were scanned at 10 °C/min from −50 to 300 °C. After cooling at 50 °C/min, an immediate rescan was run from 0 °C to 350 °C to verify the eventual presence of residual heat of reaction. For all uncured samples no peaks were observed in the second scan, therefore the Δ*H* obtained from the exothermal peak in the first scan was assumed as a measure of the total heat of reaction (Δ*H_T_*). The residual heat of reaction (Δ*H*_Res_) was determined from the measurements performed on cured samples. The measurements were performed at 10 °C/min from 0 °C to 350 °C. Δ*H* obtained from the exothermal peak of these scans was considered as a measure of the residual heat of reaction (Δ*H*_Res_).

#### 2.2.3. Thermogravimetric Analysis (TGA)

A Mettler TGA/SDTA 851 thermal analyzer was employed to carry out Thermogravimetric Analysis in air and in nitrogen atmosphere. The weight loss as a function of the temperature was recorded at 10 °C/min from 25 ° to 1000 °C.

#### 2.2.4. High-Resolution Transmission Electron Microscopy (HRTEM) Analysis

Jeol 2010 LaBa6 microscope operating at 200 kV, HRTEM was used to perform the morphological characterization. MWCNTs were dispersed in ethanol by ultrasonication for half an hour. A copper grid, holey carbon, was used to hold the dispersion during the observation.

#### 2.2.5. Electrical Properties

Dc volume conductivity was measured on disk-shaped specimens of about 2 mm thickness and 50 mm diameter to which a circular measurement electrode with a diameter of about 22 mm was applied. If the samples were above or below the percolation threshold, two different experimental set-ups were used. For samples beyond the percolation threshold, the experimental set-up was composed of a suitable shielded cell with temperature control, of multimeter Keithley 6517A with the function of voltage generator (max. ±1000 V) and voltmeter (max. ±200 V) and the ammeter HP34401A (min. current 0.1 µA). The experimental system for sample below the percolation threshold was instead composed of multimeter Keithley 6517A with functions of voltage generator (max. ±1000 V) and pico-ammeter (min. current 0.1 fA). All the systems were remotely controlled by the software LABVIEW^®^ (Labview 2018, National Instruments, Austin, TX, USA). 

#### 2.2.6. Self-Healing Efficiency

Self-healing tests were carried out on the composite materials by a continuous dynamic flexural deformation. In particular, samples having dimensions 3 × 10 × 35 mm^3^ and a V-shaped starter notch, (1 × 2 mm^2^) were analyzed by applying a sinusoidal deformation with a maximum amplitude of 0.1% at a frequency of 1 Hz. A precrack in the sample was induced by an impulsive load of about 25 N. The evolution of the mechanical modulus with time was considered as representative of the evolution of the healing process in the sample.

#### 2.2.7. Infrared Spectroscopy Characterization

Infrared spectroscopy (FTIR) tests were carried out using a Bruker Vertex 70 FTIR-spectrophotometer (Bruker Optics Inc. Billerica, MA, USA) in the range of 4000–400 cm^−1^, with a resolution of 2 cm^−1^ (32 scans collected). The infrared spectra were obtained in absorbance.

Infrared spectra of the cured epoxy resin and related nanocomposites were collected using 13 mm-diameter pellets of KBr as a carrier. Parts of the samples were finely ground to a powder and dispersed in the KBr pellets. It is worth noting that to be sure to avoid possible contributes of the adsorbed moisture in the spectral region of O–H signals, the powders of the samples were conditioned at 100 °C under vacuum for 24 h before their dispersion in the KBr pellets. Afterward, the pellets were conditioned at 100 °C under vacuum for 24 h again before the collection of the spectra.

## 3. Results and Discussion

### 3.1. Toughening of the Hosting Epoxy Matrix

The self-healing mechanism with RHB interactions in structural resins is limited by very low mobility of the chain segments in the thermosetting matrix, which prevent rearrangement of cooperative hydrogen bonding interactions. The challenge in this work was to improve the dynamic properties of the resin and reduce the rigidity of the matrix, acting on its phase composition. Therefore, the design of a material with small domains of the polymer at higher mobility, finely interpenetrated in the resin, has been considered.

### 3.2. Dynamic Mechanical Analysis (DMA)

#### 3.2.1. Hosting Epoxy Matrix

**Figure 5:**
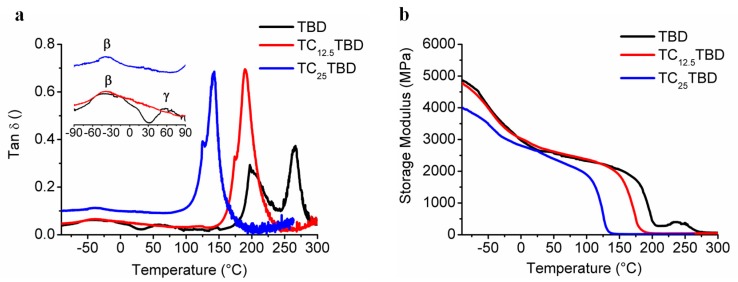
(**a**) Evolution of the loss factor (tan δ) as a function of the temperature; (**b**) Evolution of the storage modulus as a function of the temperature.

DMA investigation was considered to analyze the influence of the rubber phase on the viscoelastic response of the resin and, in particular, to assess the glass transition temperature (*T*_g_). The storage modulus, *E*’ (MPa), and the loss factor, tan δ, of the analyzed formulations at different rubber content, are shown in Figure 5.

The curve related to tan δ of the sample TBD (black curve) without rubber exhibits two distinct peaks centered at about 200 °C and 265 °C. The appearance of two peaks in the matrix has already been found in the literature [70] and is correlated to the presence of phases with different crosslinking density, for which one is characterized by enhanced segmental motion and consequently lower values of Tg. The phase composition depends on the curing degree and on the ratio between the epoxy/amine functional groups.

The addition of the liquid rubber CTBN in the epoxy matrix (samples TC12.5TBD and TC25.0TBD) determines a decrease of the *T*_g_, which appears almost as a single peak shifted at a lower temperature. The *T*_g_ decreases as the rubber phase increases. The glass transition temperatures are centered at about 190 °C for the sample TC12.5TBD and at about 140 °C for the sample TC25.0TBD. Compared to sample TBD, the relaxation mechanisms of the samples with the rubber phase are associated to only one coalesced peak that is more intense and broadened, suggesting that for these samples, the two phases characterized by different crosslinking density have converged to a single phase, which presents relaxation mechanisms in a wider interval of temperature. As shown in the inset of Figure 5a, the same trend is observed for the other two weak β and γ relaxations. The transition between −100°C and 30 °C, known as β transition, is due to crankshaft rotations of the glycidyl crosslinking segments [70,71,72], while the γ transition (between 30 °C and 120 °C) is due to unreacted molecular segments and/or inhomogeneities of the material in domains with different crosslink density. The peaks associated with these weak transitions are more pronounced for the TBD formulation and converge in a single wider peak for the rubber epoxy mixtures that present relaxation mechanisms in a range of low temperatures (−100 °C ≅ 60 °C). The lower intensity of γ transition suggests that the presence of the liquid rubber determines a reduction the material inhomogeneity and consequently of domains with different crosslink density.

It can be hypothesized that the use of the epoxy matrix toughened with the liquid rubber CNTB enhances the activation of supramolecular self-healing mechanisms for two different reasons:

- the reduced crosslink density highlighted by the decrease in the *T*_g_ values increases the chain flexibility and mobility, hence enhancing the ability of the composite to activate autorepair mechanisms;
- the modified epoxy matrix can better interact with self-healing moieties on MWCNTs due to the reduced rigidity of the matrix;

The comparison, in terms of storage modulus (Figure 5b), evidences that the values tend to decrease with increasing rubber-phase, as expected.

As observable in Figure 5b, all samples are characterized by high storage modulus values. In particular, for temperature below 100 °C, these values are higher than 2000 MPa. This result also indicates that the epoxy formulation containing the liquid rubber at a lower percentage (TC_25_TBD) has the potential to be applicable in the temperature service range of structural materials (aerospace, wind turbine, shipping, electronic industries, etc.). However, considering the *T*_g_ and the storage modulus values, the formulation containing 12.5 phr of rubber-phase has been selected as the epoxy matrix suitable to host self-healing mechanisms to be applied in aeronautical composites for which a *T*_g_ value of about 180 °C is suggested by the manufacturing industries.

#### 3.2.2. Self-Healing Epoxy Formulation

The influence of functionalized MWCNTs on the storage modulus and *T*_g_ of the self-healing nanocomposites has been investigated by DMA analysis.

**Figure 6:**
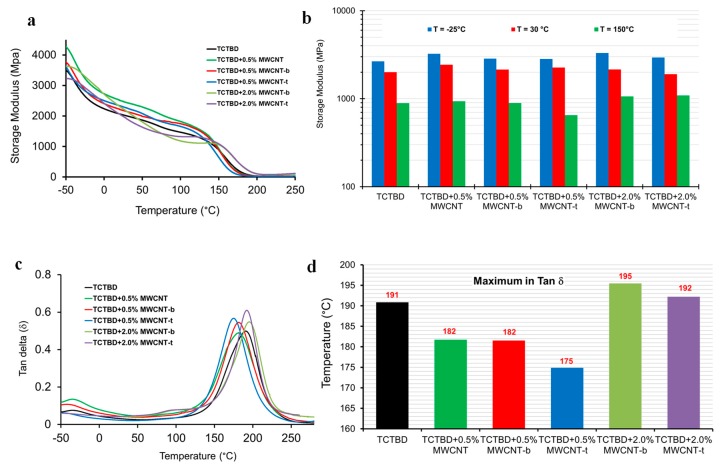
(**a**) Evolution of the storage modulus as a function of the temperature; (**b**) Values of the loss modulus at 25, 30, and 150 °C; (**c**) Evolution of the loss factor (tan δ) as a function of the temperature. (**d**) The temperature of the maximum loss factor for all nanocomposites.

Figure 6a illustrates the curves of the storage modulus (*E*’) as a function of the temperature for the samples containing different percentages of functionalized MWCNT. The graphs of the storage modulus (*E*’) of the sample containing nonfunctionalized MWCNTs and the unfilled resin have also been added for comparison. The value of the loss moduli at 25, 30, and 150 °C are also shown in Figure 6b. As expected, the storage modulus decreases with the increase of temperature. At temperatures below 10 °C, the incorporation of functionalized and nonfunctionalized nanofiller causes an increase of the storage modulus. The highest enhancement is observed for the samples containing nonfunctionalized MWCNTs and barbituric acid functionalized MWCNT-b. For temperature values between 10 °C and 140 °C, the storage modulus corresponding to the samples containing the highest percentage of functionalized MWCNTs is lower than that corresponding to the unfilled resin. Most probably, this result is caused by a reduction of the stiffness and strength of the carbon nanotubes due to the functionalization. In any case, nanocomposites containing higher percentages of functionalized MWCNTs maintain a storage modulus equal to or higher than 2000 MPa until the temperature of 30 °C is reached (see Figure 6b), whereas nanocomposites containing incorporated 0.5 wt % of functionalized MWCNTs are characterized by a storage modulus higher than 2000 MPa until 60 °C. A rapid decrease of the storage modulus is observed in the interval of temperature between 120 °C and 200 °C, corresponding to the glass transition temperature of the formulated nanocomposites, as confirmed by the loss tangent (tan δ, Figure 6c). Figure 6c shows the evolution of tan δ with the temperature. The presence of two peaks highlights two different temperatures associated with the relaxation phenomena occurring in the samples. As for the previous case, the highest temperature for the relaxation phenomena, observable between 170 and 200 °C, is due to the *T*_g_ of the epoxy matrix. The lower temperature associated with the relaxation phenomena, observable between −30 °C and −50 °C, is due to both the *T*_g_ of the rubber phase and the secondary relaxation phenomenon of the epoxy resin, as previously discussed in the literature [60]. The maximum of the tan δ peak is shown in Figure 6d for all the formulations. It shifts to higher temperatures (Figure 6c) for the formulations nanofilled with 2.0 wt % of functionalized MWCNTs. For these samples, an increase in the intensity of the relaxation phenomena in the temperature interval −50–0 °C, together with the appearance of the third peak between 75 °C and 120 °C is also observed. This last peak is probably due to an epoxy chain motion enhancement caused by the presence of functional groups on MWCNTs. For lower percentages of functionalized MWCNTs (0.5 wt.%), the peak between 75 °C and 120 °C is almost absent, but the main value of *T*_g_ is shifted to a temperature slightly lower than that of the unfilled sample (Figure 6d).

### 3.3. Thermal Properties

**Figure 7:**
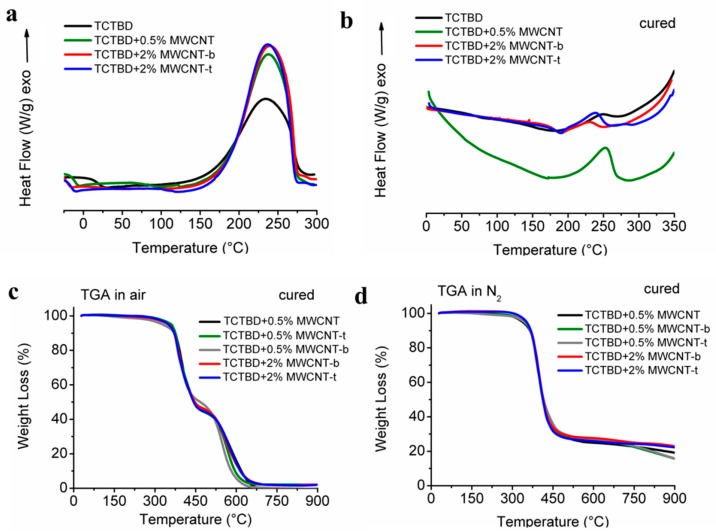
(**a**) Differential scanning colorimetry (DSC) graphic of the sample TCTBD and the samples TCTBD + 0.5% MWCNT, TCTBD + 2% MWCNT-b, TCTBD + 2% MWCNT-t; (**b**) DSC graphics of the solidified sample TCTBD and the samples TCTBD + 0.5% MWCNT, TCTBD + 2% MWCNT-b, TCTBD + 2% MWCNT-t; (**c**) TGA graphics of the cured sample TCTBD filled with nonfunctionalized MWCNTs and two different percentages of functionalized MWCNTs, in airflow; (**d**) TGA graphics of the solidified sample TCTBD filled with nonfunctionalized and two different percentages of functionalized MWCNTs, in nitrogen flow.

DSC investigation has been carried out on uncured multifunctional self-healing nanocomposites (Figure 7a) and on the same formulations after the curing (Figure 7b). The curing degree (DC) has been determined according to the procedure described in Section 2.2.2. This analysis has also been performed on the toughened sample TCTBD and on the same sample nanofilled with 0.5% of nonfunctionalized MWCNTs. The thermograms in Figure 7b indicate that all the solidified samples contain a little amount of unsolidified resin, which further cures during the temperature scan. In detail, the uncharged resin and the resin charged with both functionalized and nonfunctionalized MWCNTs show an exothermic peak between 175 °C and 275 °C due to the curing reactions. The resins nanofilled with MWCNT-b and MWCNT-t exhibit an additional small exothermic peak at a higher temperature (~280 °C). This peak is most probably due to an initial decomposition of the functional groups on the wall of MWCNTs. In fact, it is worth noting that these groups contain triazole rings or remaining azides in their structure that decompose at temperatures higher than 270 °C, causing the formation of free radicals, which can be responsible for homopolymerization reactions determining additional curing of the epoxy matrix.

Δ*H_T_*, Δ*H*_Res_, and DC values of the formulated nanocomposites are indicated in Table 2.

Sample TCTBD has a DC of 97%. The incorporation of nonfunctionalized nanotubes in the epoxy matrix results in a decrease in the DC value, which reached the value of 89% for the sample TCTBD + 0.5% MWCNT; this effect is less conspicuous for the samples with functionalized MWCNTs. In fact, both the formulations filled with 0.5% of MWCNT-b and MWCNT-t are characterized by a DC of 93%.

The curing degree increases as the amount of functionalized MWCNTs increases. For a percentage of 2.0% by weight of functionalized MWCNTs, the DC value reaches approximatively the same values of the unfilled formulation. The sample TCTBD + 2% MWCNT-b exhibits a Δ*H*_Res_ of 9.00 Jg^−1^, corresponding to the highest DC (98%) found for the self-healing formulations. Generally, for epoxy systems, the incorporation of MWCNTs at low concentration (0.32 wt.%) results in a decrease of the cross-linking density with a reduction of the curing degree; consequently, in order to drive the curing toward the same DC of the uncharged resin, higher temperatures during the curing cycle must be used [73]. In this case, the functional moieties attached to the MWCNTs allow reaching higher curing degrees, similar to that obtained for the unfilled formulation, without changing the conditions of temperature and pressure. This provides, from the perspective of possible industrial applications, an interesting energy-saving improvement.

Figure 7c,d illustrate the TGA graphics for all the solidified samples; in both air and nitrogen, for all samples, the initial thermal degradation is at about 360 °C. Sample TCTBD + 0.5% MWCNT-b, during the measurement in air flow, loses a little percentage of its weight at temperatures slightly higher than 270 °C. This seems to be in agreement with the results observed at the DSC and the hypothesis of an initial decomposition of the functional groups responsible for additional curing of the resin, as mentioned before. In any case, in both nitrogen and air atmosphere, the TGA profiles underline two distinct and well-separated thermal events. The first one, falling for all the samples in the temperature interval of 360 °C–450 °C, indicates that there is no considerable effect of MWCNTs and functional moieties on the first stage of the thermal degradation.

However, comparing the TGA in air and nitrogen flow, it is interesting to observe the values of the mass loss during this first stage of thermal decomposition. The mass loss in nitrogen (63–75%) is much higher than the mass loss in the air (47–55%), indicating that in the range of temperature below 450 °C, all the samples are more stable in air than in nitrogen. This behavior, already observed in the literature for epoxy resins, is mainly ascribed to the oxidation reactions in the gas-phase during the flaming burning of the nanofilled resins [74].

### 3.4. Transmission Electron Microscopy (TEM)

**Figure 8:**
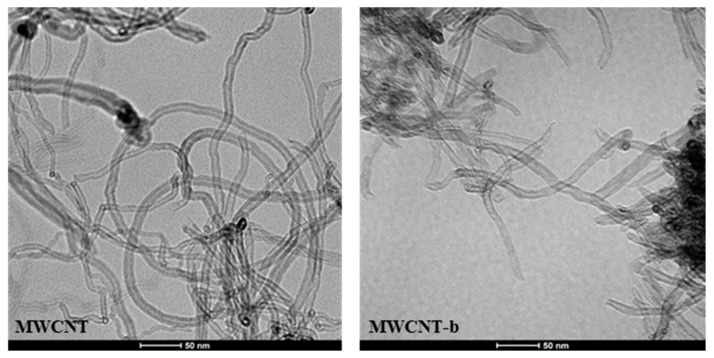
TEM images of the nonfunctionalized MWCNTs (**left**) and of the functionalized MWCNT-b (**right**).

TEM analysis has been used to better investigate the effect of covalent functionalization on MWCNTs. Figure 8 shows the comparison between the TEM image of the pristine MWCNTs (see image on the left) and the TEM image of the functionalized MWCNT-b (see image on the right). The comparison highlights that the functionalization procedure strongly affects the aspect ratio of the carbon nanofiller, attacking mainly the areas of defects and reducing the length of the MWCNTs. This effect could be responsible for the strong reduction in the values of the electrical conductivity of the self-healing nanocomposites containing embedded functionalized MWCNTs.

**Figure 9:**
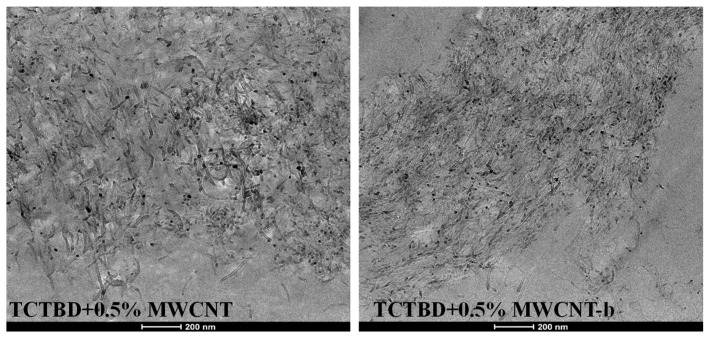
TEM images of the nanocomposites TCTBD + 0.5%MWCNT (**left**) and of TCTBD + 0.5% MWCNT-b (**right**).

Figure 9 shows the TEM images of the nanocomposites containing 0.5 wt % of nonfunctionalized MWCNT (on the left) and 0.5 wt.% of functionalized MWCNT-b (on the right).

**Figure 10:**
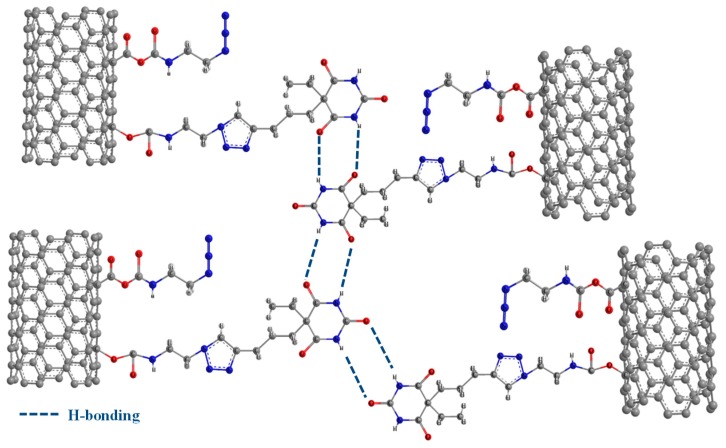
Example of moieties involved in hydrogen bonds between NH and CO functional group, for the barbiturate modified MWCNTs.

It is statistically confirmed that functionalized MWCNTs have shorter lengths than the nonfunctionalized ones. Furthermore, in the hosting matrix, the functionalized MWCNTs seem to be placed at a closer distance and constrained in such a way to be more extended compared to nonfunctionalized MWCNTs. This particular arrangement is most likely favored by the hydrogen bonding moieties on MWCNT walls (as shown in the scheme of Figure 10).

Similar morphological results have been obtained for nanocomposites containing MWCNTs-t.

### 3.5. Infrared Spectroscopy Characterization

Hydrogen bonds in the developed materials can be due to N–H⋅⋅⋅O=C, N–H⋅⋅⋅N–H, O–H⋅⋅O–H, N–H⋅⋅⋅O–H, etc. Some of these interactions, for instance, O–H⋅⋅O–H interaction or N–H⋅⋅⋅O–H interaction, are already active in the epoxy matrix (even in the absence of functional groups on the walls of carbon nanotubes). For this reason, we have evaluated the increase of hydrogen bonds specifically due to functional groups with respect to those due to the structure of the hosting epoxy matrix. This investigation has been performed by means of FT/IR analysis of the cured epoxy resin and the self-healing nanocomposites. Concerning this aspect, the attention has been focused on the absorbance of OH groups. Infrared spectroscopy can be used to analyze the O–H stretching vibration in the region between 3100 and 3650 cm^−1^. This region is diagnostic of the hydrogen bonds. Although in a solidified epoxy resin, a relevant presence of –OH groups determined by the curing process is always present [70], isolated sharp bands due to “free” hydroxyl bands are not observed because of the massive presence of O–H groups and the distances between them, which favor hydrogen bonds. Therefore, in the solidified state of an epoxy resin, the O–H stretching vibration appears as a broad band because of the hydrogen bonding formed during the curing cycle. However, the profile of the band in the region of the hydroxyl groups, and the presence of more or less accentuated shoulders together with their position can provide important information about the nature and the extent of hydrogen bonding interactions [59]. Generally, in the profile of the FT/IR spectrum, corresponding to the O–H region, a board band or a shoulder appearing at a lower frequency (between 3550–3100 cm^−1^) with respect to the signal of “free” hydroxyl band (3400–3650 cm^−1^) is diagnostic of intermolecular hydrogen bonds. It is expected that the larger the intensity of the band/shoulder, the larger the amount of hydrogen bonds and hence the larger the amount of hydrogen bond-based interactions, which affect the material properties. It is worth noting that the frequency of the hydrogen bonds dependents not only on the chemical nature of the involved atoms (N, O, etc.), but also on the distances of intramolecular bonds. Previous studies evidenced that the H⋅⋅⋅O and the N⋅⋅⋅O distances of hydrogen bonds are very sensitive to changes in the nature and environment of the donor and acceptor groups [75] and the electrostatic potential at appropriate distances [76].

These factors determine the enlargement of the band of the hydrogen bonds. For this reason, the band of the O–H group has been decomposed into different components using a complex fitting in which a Lorentzian and Gaussian contributions have been considered, as in previous papers [77,78].

**Figure 11:**
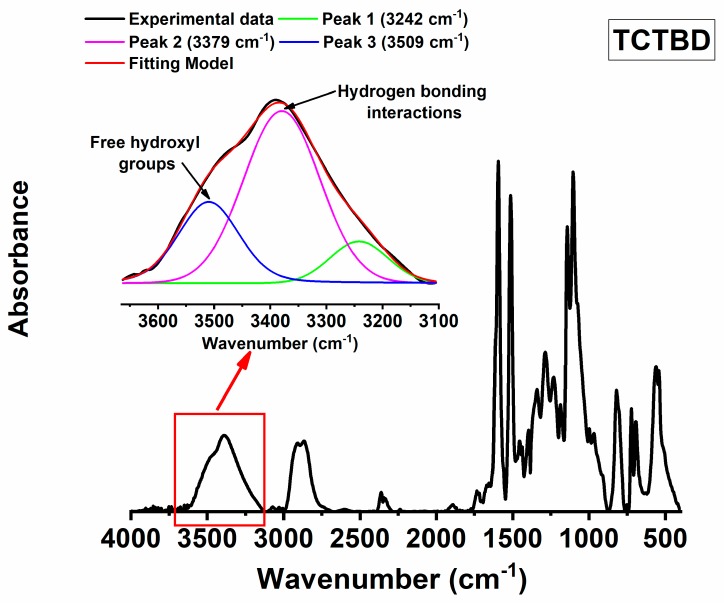
FT-IR spectrum of the TCTBD sample; deconvolution relating to the region of the OH group (in the inset).

**Figure 12:**
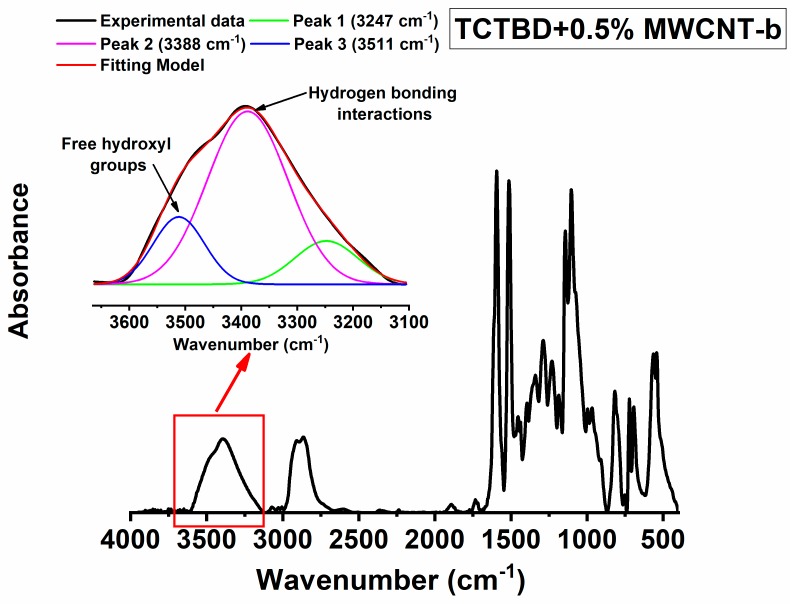
FT-IR spectrum of the TCTBD + 0.5% MWCNT-b sample; deconvolution relating to the region of the OH group (in the inset).

**Figure 13:**
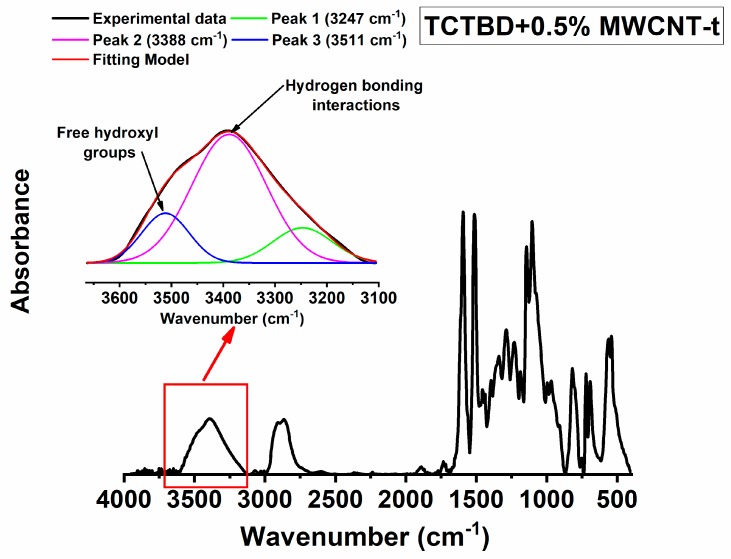
FT-IR spectrum of the TCTBD + 0.5% MWCNT-t sample; deconvolution relating to the region of the OH group (in the inset).

The results of this deconvolution procedure for the cured epoxy resin and the for the self-healing nanocomposites at a concentration of 0.5% by weight of functionalized MWCNTs, in the above-mentioned ranges of wavenumbers, are shown respectively in Figure 11, Figure 12 and Figure 13. In particular, three different peaks have been considered for this deconvolution procedure. The areas of the two peaks at about 3380 cm^−1^ (3379 cm^−1^ in the epoxy resin and 3388 cm^−1^ in the nanocomposites) and about 3245 cm^−1^ (3242 cm^−1^ in the epoxy resin and 3347 cm^−1^ in the nanocomposites) have been attributed to hydrogen bonding interactions, whereas the area of the peak at higher frequency, around 3510 cm^−1^ has been attributed to nonhydrogen-bonded or almost “free” hydroxyl groups, which can determine a band less sharp with respect to that observed in vapor phase or in very diluted solution (in nonpolar solvent) because of the solid state. The total area of the two bands at around 3380 cm^−1^ and 3245 cm^−1^ is considered to be due to hydrogen bonds.

**Figure 14:**
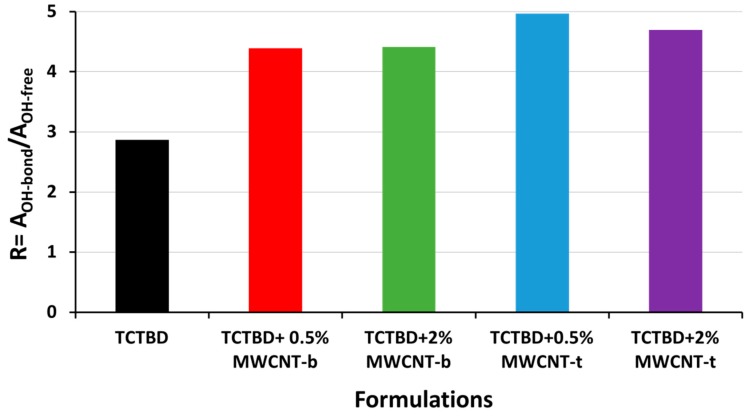
Values of the ratio R = A_OH-bond_/A_OH-free_ for the epoxy resin and the nanocomposites containing 0.5 wt % and 2% wt % of MWCNT-b and MWCNT-t.

The ratio R, between the area of the O–H bonded signal (A_OH-bond_) and the area of the free O–H signal (A_OH-free_) has been considered to make an assessment of the extent of H-bonding interactions formed in the composites. The results of this evaluation are shown in the histogram of Figure 14.

Data shown in Figure 14 clearly highlight an increase of hydrogen bonds in the samples containing incorporated functionalized MWCNTs. In particular, the ratio R = A_OH-bond_/A_OH-free_ for the self-healing nanocomposites shows an increased percentage, which ranges between 68% and 80% (depending most of all on the nature of the functional group).

### 3.6. Electrical Properties

**Figure 15:**
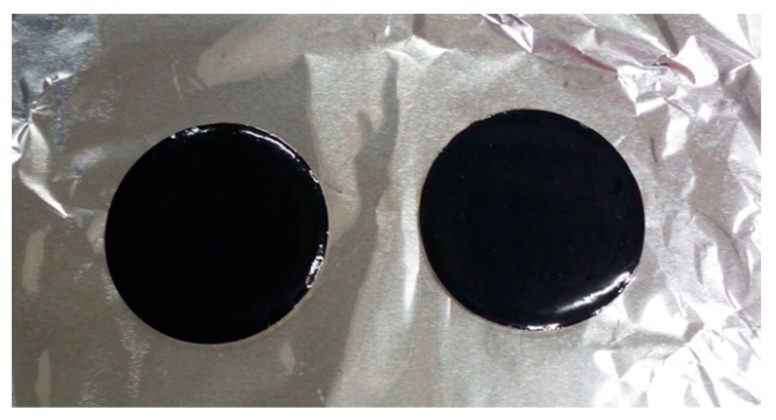
Disk-shaped specimens.

In order to evaluate the electrical behavior of nanocharged materials, electrical conductivity measurements have been performed on disk-shaped specimens shown in Figure 15. The values of the electrical conductivity are shown in Table 3.

It is evident that the incorporation of pristine MWCNTs to the resin TCTBD causes formation of conductive paths changing the behavior of the material from insulant for the TCTBD with an electrical conductivity value of 1.16 × 10^−14^ S/m to conductive for the sample TCTBD+0.5%MWCNT, having an electrical conductivity value of 2.56 × 10^−2^ S/m.

**Figure 16:**
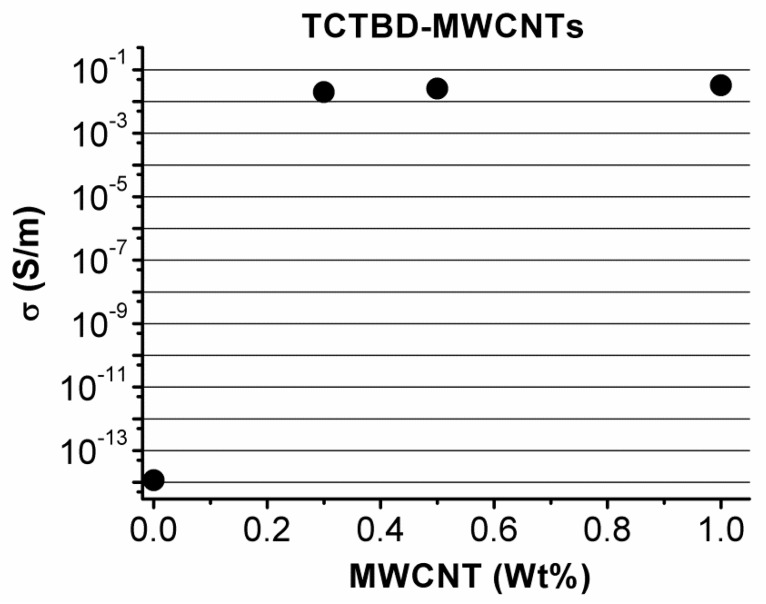
Electrical conductivity of TCTBD sample versus MWCNT weight percentage.

Figure 16 shows the percolation curve of the Epoxy/MWCNT nanocomposite systems. It is observable that at a concentration of 0.5% by weight of MWCNTs, the sample is beyond the electrical percolation threshold (EPT) and hence behaves as a conductive material.

The values of electrical conductivity shown in Table 3 indicate that the functionalization of MWCNTs has a relevant influence on the electrical behavior of the nanocomposites, reducing their electrical conductivity to values of pS/m. This result is most likely due to the modifications in the aspect ratio of the nanofiller, evidenced by Transmission Electron Microscopy (TEM) investigation, as a result of the functionalization procedure. The functionalization partially reduces the π-electron delocalization of the aromatic rings, and hence the sp^2^ hybridization of the carbon atoms, as already found in the literature for other types of covalent functionalization. In order to achieve values in the electrical conductivity of the self-healing nanocomposites comparable to those obtained with nonfunctionalized MWCNTs, nanofiller loadings up to 2.0 by wt % (see Table 2) are necessary. It is worth noting that a percentage of 2% by weight of functionalized MWCNTs corresponds to a percentage of 1.5% by weight of MWCNTs, calculated by subtracting the weight of the functional groups. 

### 3.7. Self-Healing Efficiency

**Figure 17:**
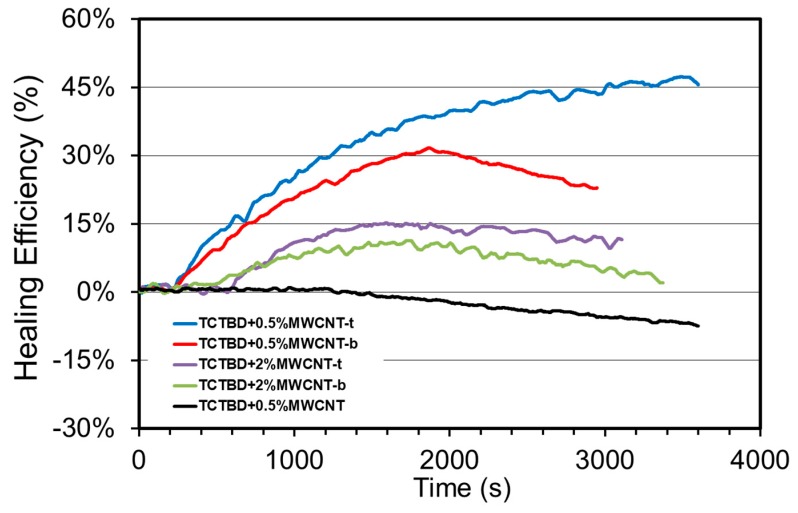
Time evolution of the healing efficiency for all the formulations.

As described in Section 2.2.6, for all the samples, the evolution of the storage modulus during a dynamic test at constant temperature was considered as a measurement of the healing efficiency. In particular, the healing efficiency (Hr) is defined here as the ratio between the actual elastic modulus (*E*’) during the measurement and the elastic modulus of the pristine sample (*E*’) before applying the crack. Figure 17 shows the healing efficiency of all the analyzed samples as a function of the time during the test. As clearly shown, all the samples containing modified MWCNTs present partial recovery of the elastic modulus during monitoring. The behavior is quite similar for all formulations. After an initial delay, the recovery mechanism is evidenced by a rise of the modulus up to a maximum and a subsequent reduction of the healing efficiency. This decrease of the healing efficiency after a maximum has previously been found in the literature [79] and it has been ascribed to a crack propagation as a result of the cyclic fatigue stress. The sample containing nonfunctionalized carbon nanotubes, TCTBD + 0.5%MWCNT, did not show any healing mechanisms, demonstrating that MWCNTs alone are not able to confer self-healing functionality to the epoxy resin.

It is worth noting that, contrary to the expected results, the higher the percentage of functionalized MWCNTs, the lower the entity in the recovery mechanisms. This result is most likely due to the higher rigidity and therefore the reduced dynamic mobility of the epoxy matrix containing the highest percentage of functionalized MWCNTs. In fact, TCTBD + 2.0%MWCNT-b and TCTBD + 2.0% MWCNT-t, respectively, undergo glass transition at 195 °C and 192 °C, whereas, for the same samples with a content of functionalized MWCNTs of 0.5%, the values of glass transition temperature are lower: 182 °C for TCTBD + 0.5%MWCNT-t and 175 °C TCTBD + 0.5% MWCNT-b (see Figure 6d).

## 4. Conclusions

In this work, electrically conductive self-healing resins based on RHB interactions have been developed. The change in the aspect ratio of MWCNTs due to the functionalization procedure requires higher MWCNT percentages (2% by weight) to reach the same value in the electrical conductivity achievable with nonfunctionalized MWCNTs.

This paper demonstrates the validity of the hydrogen bonding strategy where the dynamic repetition of hydrogen bonds is enabled, as an effective solution to confer self-healing ability in addition to the ability to contrast the electrical insulating property typical of this kind of material. Occurring stress can cause the breaking of hydrogen bonds, which have the ability to regenerate the electron donor/acceptor interaction without any external stimulus. The approach proposed here offers combined advantages that allow the design and manufacturing of self-healing nanocomposites with potential for fulfilling many requirements of structural applications in many technological fields, such as aerospace, automotive, etc. The chosen functional groups, covalently attached on the walls of carbon nanotubes, are able to activate hydrogen bonds via electron donation and electron acceptance, as shown in Figure 10 for MWCNTs functionalized with barbiturate. The peculiar chemical formulation allows RHB interactions between the functional groups on different MWCNTs, or between the functional groups on MWCNTs and the modified rubber phase and the epoxy matrix hardened with DDS. A CuAAC “click” reaction was used to perform the functionalization of the MWCNTs with hydrogen bonding moieties. It is well recognized that this kind of reaction presents excellent yields with a high tolerance of functional groups. In this paper, it has been deduced that the electrical properties of the self-healing nanocomposites can be suitably tailored by controlling the percentage of MWCNTs. In particular, for self-healing nanocomposites containing 2.0% by weight of MWCNT-b and MWCNT-t, electrical conductivity values of 6.76 × 10^−3^ S/m and 3.77 × 10^−2^ S/m have been obtained, respectively. A lower amount of functionalized MWCNTs leads to stronger recovery mechanisms. This result is most likely due to the lower rigidity of the epoxy matrix containing the lower amount of functionalized MWCNTs. In fact, the glass transition temperatures of samples TCTBD + 2.0%MWCNT-b and TCTBD + 2.0%MWCNT-t are 195 °C and 192 °C, respectively, whereas, for the same samples containing a lower percentage of functionalized MWCNTs, the glass transition temperatures are 182 °C and 175 °C. Considering the results of electrical conductivity shown in Figure 16, further experiments are in progress with the aim of obtaining higher values of electrical conductivity and self-healing efficiency. The simultaneous use of nonfunctionalized and functionalized MWCNTs has the potential to allow reaching the EPT at a very low content of MWCNTs, hence preserving the maximum healing efficiency of the samples. The curing degree, glass transition temperatures, and storages moduli of the formulated samples are typical for functional structural material applications.

## Figures and Tables

**Table 1:** Self-healing formulations.

Sample	MWCNT Type	MWCNT [%]	CTNB [phr]
TCTBD	MWCNT	0	12.5
TCTBD + 0.5% MWCNT	MWCNT	0.5	12.5
TCTBD + 0.5% MWCNT-b	MWCNT-b	0.5	12.5
TCTBD + 0.5% MWCNT-t	MWCNT-t	0.5	12.5
TCTBD + 2% MWCNT-b	MWCNT-b	2.0	12.5
TCTBD + 2% MWCNT-t	MWCNT-t	2.0	12.5

**Table 2:** Results of the DSC analysis.

Sample	Cure Degree DC [%]	Δ*H*_Res_ [Jg^−1^]	Δ*H_T_* [Jg^−1^]
TCTBD	97	8.16	283.42
TCTBD + 0.5% MWCNT	89	48.48	429.32
TCTBD + 0.5% MWCNT-b	93	25.21	365.56
TCTBD + 0.5% MWCNT-t	93	30.63	415.64
TCTBD + 2% MWCNT-b	98	9.00	437.87
TCTBD + 2% MWCNT-t	95	21.22	451.45

**Table 3:** Values of electrical conductivity for the analyzed samples.

Sample	% MWCNT	Electrical Conductivity [S/m]
TCTBD		1.16 × 10^−14^
TCTBD + 0.5%MWCNT	0.5	2.56 × 10^−2^
TCTBD + 0.5%MWCNT-b	0.38	6.28 × 10^−12^
TCTBD + 0.5%MWCNT-t	0.38	6.47 × 10^−12^
TCTBD + 2.0%MWCNT-b	1.50	6.76 × 10^−3^
TCTBD + 2.0%MWCNT-t	1.50	3.77 × 10^−2^
